# Navigating the complexities of severe Cushing’s syndrome in the ICU: a case report

**DOI:** 10.3389/fmed.2026.1817935

**Published:** 2026-05-11

**Authors:** Kunchala Anand, Velmurugan Selvam, M. K. Renuka

**Affiliations:** Sri Ramachandra Institute of Higher Education and Research, Chennai, India

**Keywords:** ACTH-dependent Cushing’s syndrome, acute bacterial meningitis, block and replace therapy, etomidate, severe Cushing’s syndrome

## Abstract

Severe Cushing’s syndrome (SCS) is a rare but potentially fatal endocrine emergency. We describe a woman in her fifties with a history of pituitary macroadenoma who presented with mood disturbance, proximal muscle weakness, and Cushingoid features. On admission to the intensive care unit (ICU) she was febrile, hypoxic, hypotensive, and disoriented, with imaging prior confirming recurrent pituitary macroadenoma and biochemical tests showing severe hypercortisolism. She was managed with intravenous etomidate, starting with a bolus followed by titrated infusion, which achieved rapid cortisol suppression. A block-and-replace strategy was then implemented, introducing physiological hydrocortisone once cortisol levels fell to the target range. Broad spectrum antimicrobial therapy and organ support were provided. Although biochemical control was achieved, her course was complicated by ventilator-associated pneumonia with multidrug-resistant organisms, culminating in refractory septic shock and death. This case underlines the diagnostic challenges of recognizing SCS in sepsis and the clinical value of etomidate-based block-and-replace therapy.

## Introduction

Cushing’s syndrome is a condition that is brought on by chronic hypercortisolaemia through an excess of exogenous or endogenous cortisol exposure. Endogenous forms show, a female predilection, peak incidence in the fourth decade of life, and about 80% dependence on adrenocorticotropic hormone (ACTH). Its clinical picture spans variations from a mild, pernicious presentation to rapidly progressing, severe, fulminating forms (1–3).

Severe Cushing’s syndrome (SCS) is a life-threatening condition marked by rapidly rising cortisol levels and is frequently associated with metabolic derangements, psychiatric symptoms, opportunistic infections, sepsis, and cardiovascular compromise that contribute to its aggressive course and high mortality rate (4, 5).

While surgical resection is the definitive treatment, rapid normalization of cortisol concentrations is paramount and can be achieved using adrenal steroidogenesis inhibitors such as etomidate, ketoconazole, and metyrapone, while parallelly addressing the comorbidities present (2, 6, 7).

In critically ill patients, etomidate is intravenously administered due to its rapid action and relatively stable safety profile. Etomidate, an imidazole derivative, suppresses cortisol synthesis via inhibition of adrenal 11β-hydroxylase and is employed in a “block and replace therapy.” The “block and replace” regimen that consists of complete suppression with etomidate followed by physiological replacement with hydrocortisone is often the preferred strategy for hypercortisolism due to an excess ectopic ACTH (3, 7–9).

The present report describes a patient with recurrent pituitary macroadenoma who developed SCS complicated by severe sepsis. It illustrates both the diagnostic overlap between SCS and infection and the therapeutic role of etomidate in the ICU (intensive care unit), while also showing how late recognition contributed to an unfavorable outcome.

## Case description

A woman in her mid-fifties with known type 2 diabetes, hypertension, and hypothyroidism was on oral ketoconazole and regular endocrine follow-up for a pituitary macroadenoma, which she had undergone trans-nasal trans-sphenoidal resection for in 2010, however, detailed histopathological and immunohistochemical records from the initial surgery were not available at the time of presentation.

Following surgery, she developed hypothyroidism and remained under regular endocrine follow-up. However, documentation regarding comprehensive evaluation of the hypothalamic–pituitary–adrenal (HPA) axis, including formal assessment of cortisol reserve, was unavailable. Prior to the current presentation, she had been initiated on oral ketoconazole (200 mg twice daily) as part of medical management for suspected recurrent hypercortisolism (10). However, detailed records of the initial biochemical workup, including confirmatory tests such as dexamethasone suppression testing or 24-h urinary free cortisol, were not available.

Despite ongoing therapy, the patient continued to exhibit progressive clinical and metabolic features suggestive of active hypercortisolism, indicating suboptimal disease control. She had visited the outpatient department (OPD) 4 days prior to admission in the ICU with a complaint of fatigue, recurrent headaches, mood changes and crying spells, weakness in her arm and legs, and swelling of the lower limbs; all in increasing frequency and intensity over a period of a few months. Her accompanying relatives confirmed noticing the frequent mood swings and general social withdrawal in the preceding months.

**FIGURE 1:**
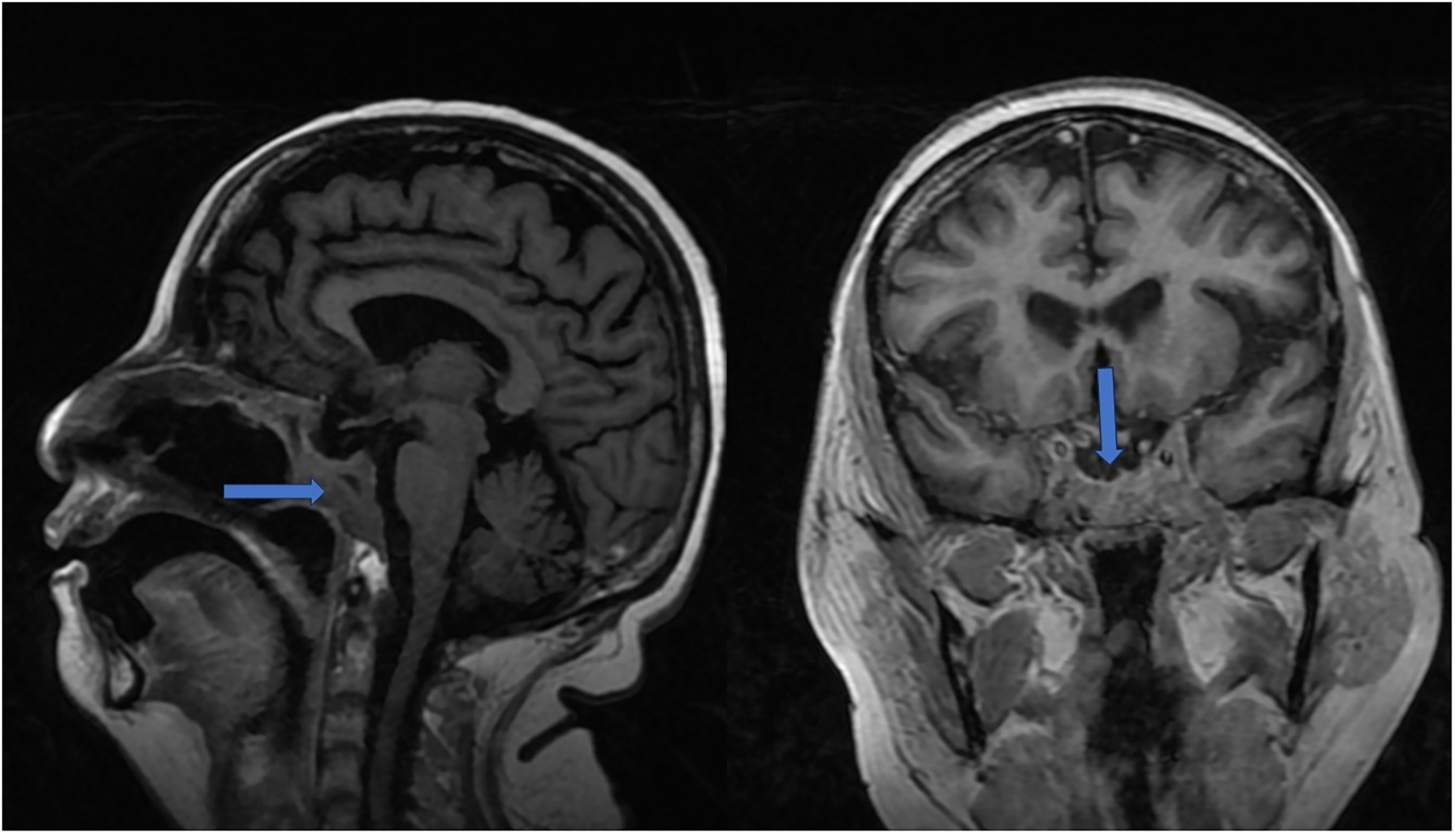
Sagittal T1-weighted MRI showing pituitary macroadenoma/Coronal T1-weighted MRI showing pituitary macroadenoma with sphenoid sinus extension.

On examination at the outpatient department, she displayed classical Cushingoid features, including a rounded, plethoric facies; bilateral proptosis; a dorsocervical fat pad; thin, fragile skin; and proximal myopathy. These findings prompted an MRI of the brain, which revealed an irregular, enhancing lesion measuring 3.4 × 4.6 × 4.5 cm, extending into the infrasellar region with an empty sella, suggestive of a recurrent pituitary macroadenoma (Figure 1). She was referred for a neurological assessment and scheduled for resection. However, within 4 days of her visit to the OPD, before her surgery could be scheduled, she developed a cough, high fever with delirium, and worsening breathlessness, for which she was brought to the hospital.

Initial physical assessment revealed that she was febrile, tachypnoeic, hypoxic, hypotensive and disoriented with a score of 8/15 on the Glasgow Coma Scale (GCS). Endotracheal intubation was carried out, and she was admitted to the ICU where mechanical ventilation was initiated.

### Timeline

Table 1 summarizes the clinical events from the date of presentation at the OPD (Day 4) leading up to cardiopulmonary arrest (Day 12).

**TABLE 1:** Timeline illustrating major clinical events, including presentation, diagnosis of methicillin-sensitive *Staphylococcus aureus* (MSSA) meningitis, progression of hypercortisolism, etomidate therapy, block-and-replace initiation, ventilator-associated pneumonia (VAP) onset, and subsequent deterioration leading to death.

Day	Event	Details
Day 4 (4 days prior to presentation to the emergency department)	Symptom onset	Complained of fatigue, recurrent headaches, mood swings, and weakness in arms and legs. MRI suggestive of recurrent pituitary macroadenoma.
Day 1 – admission to ICU	Presented to emergency department with acute symptom (fever, cough, breathlessness, altered mental state)	Patient admitted in ICU
	Hypokalemia	IV potassium supplementation
	Severe hyperglycemia with DKA	Insulin therapy initiated
	Lumbar puncture performed	CSF suggested bacterial meningitis (Gram-positive cocci). Empirical IV antimicrobials (ceftriaxone, vancomycin) initiated.
Day 3	CSF culture positive for MSSA	IV antibiotics de-escalated to cefazolin
	Worsening biochemical hypercortisolism; refractory electrolyte imbalance (K^+^, PO_4_^3–^, Mg^2 +^)	Etomidate infusion initiated (0.01 mg/kg/h); prophylactic antibiotics started
Day 6	Persistent hypercortisolism	Etomidate titrated to 0.1 mg/kg/h
Day 7	Serum cortisol dropped to 18 μg/dL (<500 nmol/L)	Block-and-replace therapy initiated
Day 10	Suspected ventilator-associated pneumonia (VAP)	Etomidate infusion discontinued (after 7 days)
Day 12	VAP microbiology confirmed	Ventilator-associated pneumonia due to multidrug-resistant pathogens; clinical deterioration due to sepsis progressing to MODS
Day 13	Cardiopulmonary arrest	Death

### Sequence of events

Preliminary blood reports revealed severe hyperglycemia and leucocytosis with raised inflammatory markers; chest imaging demonstrated consolidation in the left lower lobe that suggested a possible severe, community-acquired pneumonia with septic encephalopathy.

A lumbar puncture followed by Cerebrospinal fluid examination pointed toward acute bacterial meningitis (Table 2).

**TABLE 2:** Results of the cerebrospinal fluid (CSF) analysis with reference ranges and interpretation.

Parameter	Patient value	Reference range	Interpretation
Opening pressure	28 cm H2O	6–20 cm H2O	Elevated
Total leukocyte count	224 cells/μL	0–5 cells/μL	Markedly elevated
Differential count	52.2% neutrophils	<2%	Neutrophilic predominance
Protein	78.4 mg/dL	15–45 mg/dL	Elevated
Glucose (CSF)	10 mg/dL	45–80 mg/dL	Markedly reduced
Serum glucose	280 mg/dL	70–140 mg/dL (random)	Elevated
CSF/serum glucose ratio	0.04	>0.6	Severely reduced
Lactate	81.5 mg/dL	10–22 mg/dL	Markedly elevated
Gram stain	Gram-positive cocci	NA	S. bacterial meningitis

Empirical broad-spectrum antimicrobial therapy was initiated, including antibacterial, antiviral, and antifungal coverage, along with Pneumocystis jirovecii pneumonia (PCP) prophylaxis, in line with institutional protocol for patients with severe hypercortisolism and immunosuppression.

Supportive management included aggressive intravenous potassium supplementation for her persisting hypokalemia, continuous insulin infusion for glycaemic control, vasopressor therapy as indicated, and pharmacological prophylaxis for deep venous thrombosis. Despite these interventions, she experienced repeated episodes of Diabetic ketoacidosis (DKA).

Bacterial culture of the cerebrospinal fluid (CSF) confirmed methicillin-sensitive *Staphylococcus aureus* (MSSA), establishing the diagnosis of acute bacterial meningitis. Blood cultures also grew the same organism, indicating concurrent bacteremia. Based on culture sensitivity results, antibiotic therapy was appropriately de-escalated to intravenous cefazolin.

An endocrine assessment was conducted, given her overt Cushingoid features and metabolic disturbances. An endocrine analysis (Table 3), along with repeated episodes of DKA and persistent hypokalemia (2.5–3.0 mmol/L) despite aggressive replacement, further supported our diagnosis of severe Cushing’s syndrome.

**TABLE 3:** Results of endocrine analysis with reference ranges and interpretation.

Parameter	Patient value	Reference range	Interpretation
Morning serum cortisol (total)	>63.4 μg/dL	5–25 μg/dL	Markedly elevated
Plasma ACTH	80.9 pg/mL	10–60 pg/mL	Elevated
Serum potassium	2.5–3.0 mmol/L (persistent)	3.5–5.0 mmol/L	Severe hypokalemia

**FIGURE 2:**
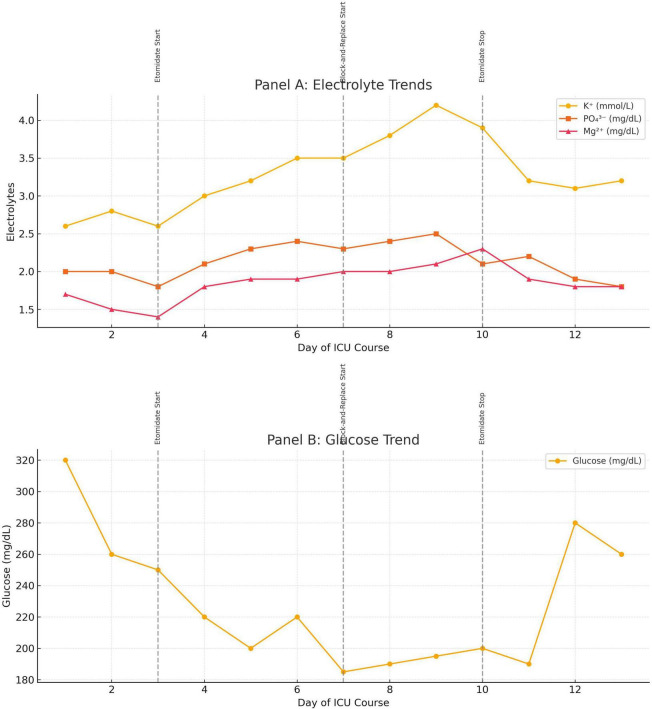
**(A)** Trends in serum electrolytes (potassium, phosphate, magnesium) during the ICU course, demonstrating initial refractory abnormalities with subsequent improvement following endocrine-directed therapy. **(B)** Corresponding serum glucose levels, showing rapid decline after initiation of etomidate and block-and-replace therapy, with later rise during sepsis-related deterioration. Vertical dashed lines denote timing of key interventions: initiation of etomidate infusion (Day 3), commencement of block-and-replace therapy (Day 3 and Day 7), and discontinuation of etomidate (Day 10).

**FIGURE 3:**
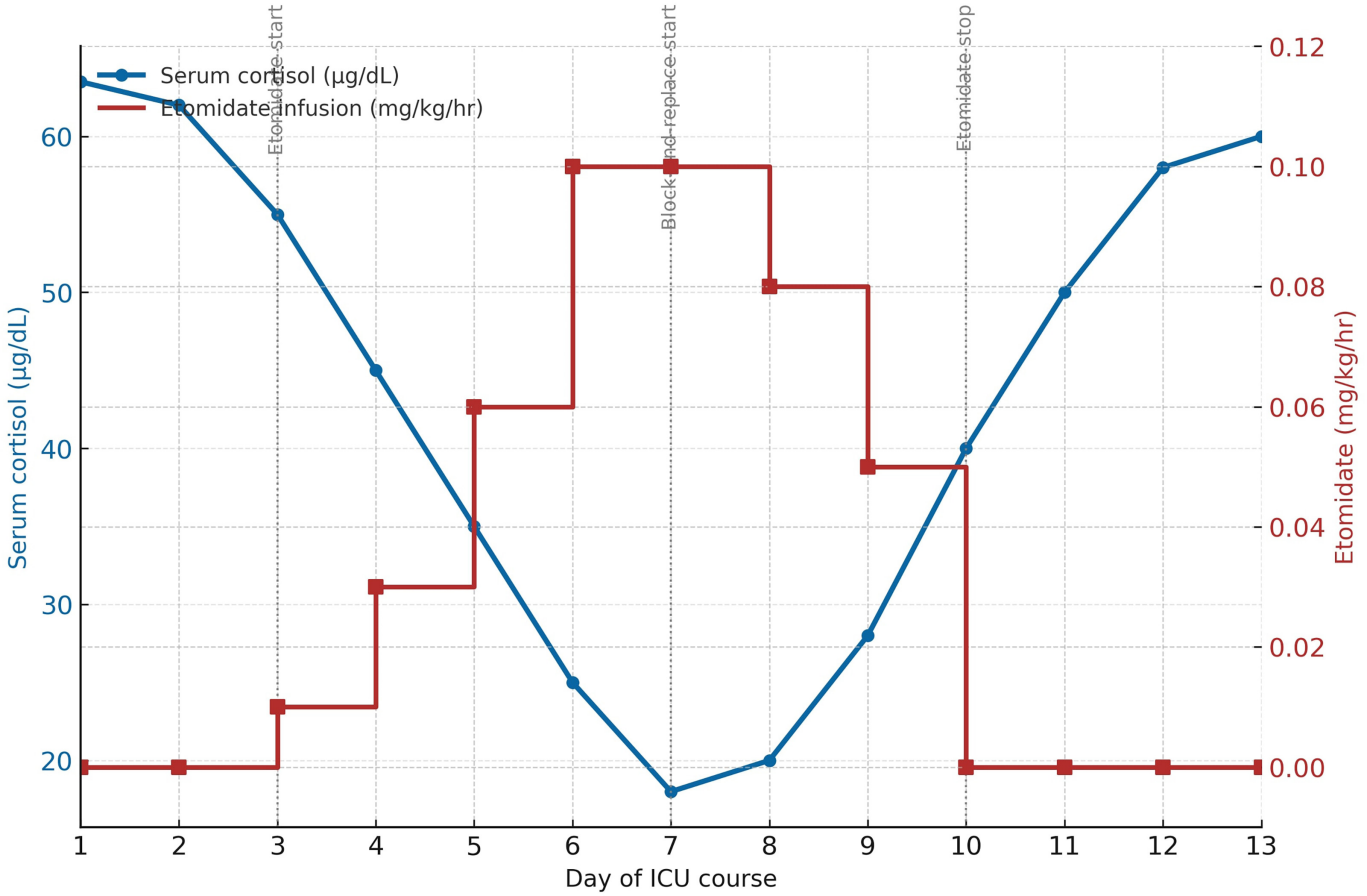
Dual-axis plot demonstrating the relationship between serum cortisol concentrations and etomidate infusion during the patient’s ICU course. The admission cortisol level was markedly elevated (>63.4 μg/dL), consistent with severe hypercortisolism. Following initiation of etomidate infusion on Day 3, cortisol levels declined progressively, reaching a nadir (~18 μg/dL) by Day 7 after commencement of block-and-replace therapy. Etomidate was discontinued on Day 10. A subsequent rise in cortisol values corresponded with evolving sepsis and clinical deterioration. Dashed vertical lines denote the timing of key endocrine interventions.

Due to her inability to tolerate oral steroidogenesis inhibitors, intravenous block and replace therapy was initiated using a 5 mg bolus of etomidate, followed by an infusion starting at 0.01 mg/kg/h, which proved ineffective in controlling her serum cortisol levels. The etomidate dose was increased to 0.1 mg/kg/h, following which serial cortisol measurements demonstrated a steady decline, with levels falling below 30 μg/dL within 3 days. Target cortisol was 15–20 μg/dL to avoid adrenal crisis in the setting of sepsis; once serum cortisol dropped to 18 μg/dL (<500 nmol/L), intravenous hydrocortisone was introduced at physiological replacement doses (50 mg IV every 6 h) on Day 7, as part of block and replace therapy. Figure 2 depicts the electrolyte and glucose trajectory, and Figure 3 plots the etomidate dose- response curve.

Given her clinical instability, and as pharmacological management was successful in regulating her serum cortisol, immediate neurosurgical intervention was deferred and planned as elective surgery after adequate stabilization.

Despite the partial metabolic and hemodynamic improvements observed around Days 6–8, her condition worsened due to the development of suspected ventilator-associated pneumonia from multi-drug-resistant pathogens and a secondary fungal infection, necessitating the discontinuation of etomidate.

During the course of hospitalization, the patient developed carbapenem-resistant Pseudomonas aeruginosa bacteremia, along with a secondary pulmonary infection due to Elizabethkingia species, which was susceptible only to rifampicin and minocycline. In addition, urine cultures grew Candida tropicalis.

Despite initiation of targeted antimicrobial therapy guided by culture and sensitivity reports, along with appropriate systemic antifungal therapy and continuation of antiviral prophylaxis, the patient’s clinical status continued to deteriorate. She progressed to refractory septic shock, requiring escalating vasopressor support, and subsequently developed multiorgan dysfunction syndrome (MODS).

The patient succumbed approximately 2 weeks after ICU admission. The immediate cause of death was attributed to polymicrobial sepsis, occurring in the background of severe ACTH-dependent hypercortisolism, which likely contributed to significant immunosuppression and poor therapeutic response.

## Discussion

Cortisol excess profoundly weakens host defenses by suppressing both innate and adaptive immunity, attenuating fever, impairing neutrophil activity, and limiting lymphocyte function. As a result, patients often develop severe or opportunistic infections. These infections, particularly those caused by multidrug-resistant organisms, remain the major cause of death in SCS. This case illustrates the diagnostic and therapeutic challenges of SCS in the ICU.

Diagnostic overlap with sepsis: The clinical presentation in this patient initially suggested severe infection, with fever, altered mental status, and pulmonary consolidation on imaging, further corroborated by cerebrospinal fluid culture confirming MSSA meningitis. However, ongoing metabolic derangements—particularly refractory hypokalemia and recurrent episodes of DKA were not adequately explained by infection alone. The coexistence of these abnormalities with classical Cushingoid stigmata prompted further endocrine evaluation. Cortisol excess is known to blunt febrile and inflammatory responses by impairing neutrophil chemotaxis, reducing lymphocyte activity, and altering cytokine signaling (2, 10). As a result, infections in SCS may present atypically or appear disproportionately severe, delaying recognition of the underlying endocrine disorder. Rubinstein et al. demonstrated that such diagnostic delays significantly increase mortality in patients with Cushing’s syndrome (11).

Sepsis as a central complication: Infections remain the leading cause of death in SCS (2, 11). Opportunistic and multidrug-resistant pathogens, including bacteria, fungi, and viruses, are frequently implicated (11). Current guidelines recommend the early initiation of broad-spectrum antibacterial agents, antifungal therapy, antiviral prophylaxis when appropriate, and routine Pneumocystis jirovecii prophylaxis in patients with severe hypercortisolism (8, 10). Despite adherence to these measures, our patient developed ventilator-associated pneumonia due to resistant organisms, illustrating that infection risk cannot be fully mitigated in the context of profound immunosuppression.

Therapeutic considerations: The cornerstone of SCS management is urgent hypocortisolaemic therapy. Oral steroidogenesis inhibitors such as ketoconazole and metyrapone may be used in stable patients, but they are impractical in critically ill individuals. Etomidate remains the only available intravenous option that can provide rapid and titratable suppression of cortisol synthesis through adrenal 11β-hydroxylase inhibition (9, 12, 13). In this case, etomidate infusion achieved effective biochemical control, and once serum cortisol levels approached the physiological range, hydrocortisone was introduced as part of a block-and-replace regimen (8, 11). This strategy is widely regarded as safe and effective in unstable patients, provided close ICU monitoring is undertaken to titrate dosing, prevent over suppression, and detect adverse effects such as adrenal insufficiency or sedation. A recent systematic review and meta-analysis further confirmed the efficacy and safety of etomidate in severe Cushing’s syndrome (9).

In block-and-replace therapy, the choice of glucocorticoid warrants consideration. Hydrocortisone is commonly preferred due to its physiological profile and ease of titration, particularly in critically ill patients. However, it may interfere with serum cortisol assays due to cross-reactivity. Dexamethasone, which does not significantly cross-react, allows more accurate biochemical monitoring. Despite this, hydrocortisone was selected in this case to provide physiological replacement and enable flexible dose adjustment in the setting of sepsis, highlighting the need to balance analytical accuracy with clinical practicality (10).

Persistence of infection risk despite biochemical control: Although cortisol levels were successfully normalized in this patient, her clinical condition deteriorated due to overwhelming sepsis. Immunological recovery lags behind biochemical improvement, which leaves patients vulnerable to opportunistic and hospital-acquired infections even after hypercortisolism is controlled. Similar outcomes have been documented in previous reports, where infection rather than hypercortisolism itself was the proximate cause of death (10, 12).

Surgical considerations: Definitive treatment of Cushing’s syndrome requires surgical resection of the underlying lesion. In the severe form, however, hemodynamic instability often makes urgent surgery unsafe. Etomidate therefore acts as an effective bridging measure, allowing temporary control of cortisol excess until elective surgery can be safely performed (2, 10). In this case, reoperation for recurrent pituitary macroadenoma was considered once the patient stabilized, but deterioration due to sepsis ultimately prevented definitive intervention.

Prognostic implications: The timing of diagnosis and initiation of therapy is a major determinant of outcome in SCS. By the time of ICU admission, this patient already had advanced hypercortisolism and severe infection. Even with recognized best practices—including etomidate infusion, block-and-replace therapy, and broad-spectrum antimicrobial prophylaxis—the disease trajectory proved irreversible. Rubinstein et al. showed that delays in diagnosis significantly increase mortality in Cushing’s syndrome (11), while Marques and Boguszewski highlighted the importance of not only achieving biochemical control but also implementing aggressive, early management of sepsis and related complications (10).

## Conclusion

Severe Cushing’s syndrome is a rare endocrine emergency that may closely mimic severe sepsis at presentation, particularly in the intensive care setting. The coexistence of refractory hypokalemia, recurrent DKA, and classical Cushingoid features should prompt consideration of underlying hypercortisolism, especially when metabolic abnormalities appear disproportionate to infection alone. Intravenous etomidate administered within a block-and-replace regimen provides effective and titratable cortisol control in unstable patients; however, infectious complications remain the principal determinant of outcome despite biochemical improvement. Early recognition and timely endocrine-directed therapy may therefore be critical in influencing prognosis (14).

## Data Availability

The raw data supporting the conclusions of this article will be made available by the authors, without undue reservation.
